# PBMC-engrafted humanized mice models for evaluating immune-related and anticancer drug delivery systems

**DOI:** 10.3389/fmolb.2024.1447315

**Published:** 2024-08-20

**Authors:** Yoshie Kametani, Ryoji Ito, Yoshiyuki Manabe, Jerzy K. Kulski, Toshiro Seki, Hitoshi Ishimoto, Takashi Shiina

**Affiliations:** ^1^ Department of Molecular Life Science, Division of Basic Medical Science, Tokai University School of Medicine, Isehara, Japan; ^2^ Institute of Advanced Biosciences, Tokai University, Hiratsuka, Japan; ^3^ Central Institute for Experimental Medicine and Life Science (CIEM), Kawasaki, Japan; ^4^ Department of Chemistry, Graduate School of Science, Osaka University, Osaka, Japan; ^5^ Faculty of Health and Medical Sciences, School of Biomedical Science, The University of Western Australia, Crawley, WA, Australia; ^6^ Department of Internal Medicine, Division of Nephrology, Endocrinology, and Metabolism, Tokai University School of Medicine, Isehara, Japan; ^7^ Department of Obstetrics and Gynecology, Tokai University School of Medicine, Isehara, Japan

**Keywords:** humanized mouse, anticancer drug, immune system, drug delivery system, hematopoietic stem cells, peripheral blood mononuclear cells

## Abstract

Immune-related drug delivery systems (DDSs) in humanized mouse models are at the forefront of cancer research and serve as bridges between preclinical studies and clinical applications. These systems offer unique platforms for exploring new therapies and understanding their interactions with human cells and the immune system. Here, we focus on a DDS and a peripheral blood mononuclear cell (PBMC)-engrafted humanized mouse model that we recently developed, and consider some of the key components, challenges, and applications to advance these systems towards better cancer treatment on the basis of a better understanding of the immune response. Our DDS is unique and has a dual function, an anticancer effect and a capacity to fine-tune the immune reaction. The PBL-NOG-hIL-4-Tg mouse system is superior to other available humanized mouse systems for the development of such multifunctional DDSs because it supports the rapid reconstruction of an individual donor’s immunity and avoids the onset of graft-versus-host disease.

## 1 Introduction

Human cancers are characterized by cellular DNA damage and genomic instability, producing neoantigens that induce an immune response associated with chronic inflammation and changes in immunological conditions (Mardis, 2019). Although new drug delivery systems (DDSs) and immunotherapy methods have revolutionized cancer treatment (Wang et al., 2023; Yadav et al., 2024), their efficacy remains limited in most clinical settings because of a lack of information about the induced antitumor responses (Zhao et al., 2019). In addition, the development of immune-related drugs or DDSs in experimental animals is difficult because their immunity is evolutionarily distant from that of humans (Mestas and Hughes, 2004; Kohu et al., 2008), and the immune reaction is usually species-specific. Nevertheless, humanized immune mouse systems with introduced genes, engrafted cells, and various molecules of the human immune system have been developed to partially mimic the immune environment of cancer patients (Kametani et al., 2006; Kametani et al., 2016; Kametani et al., 2019; Chen et al., 2022). However, the engraftment of human lymphocytes and myeloid cells into transgenic and humanized mice has limitations and unexpected problems. This is not surprising given that the genomic and genetic organization of the human and mouse immune systems are largely different (Shiina et al., 2017). In this mini-review, we focus on a DDS and PBMC-engrafted humanized mouse model that we recently developed and consider some of the key components, challenges, and applications that can advance the two systems towards improved cancer therapies.

## 2 Immune humanized mice

### 2.1 Category of immune humanized mice models

**FIGURE 1:**
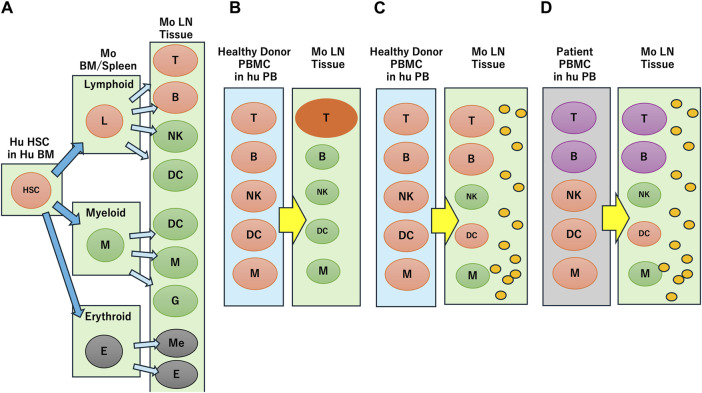
Reconstruction of human immune profiles in immune humanized mouse. **(A)** Human hematopoietic stem cell (HSC) transplantation. In mouse (Mo) bone marrow (BM), spleen and lymph node (LN) tissue, human lymphoid and myeloid cells develop into various blood cells, but erythroid cells are not engrafted. **(B)** Healthy donor peripheral blood mononuclear cell (PBMC) transplantation. Only T cells are activated through xenogeneic reaction. **(C)** Transplantation of healthy donor PBMC in the presence of human IL-4 (indicated by small orange circles). T cells are not activated through xenogeneic reaction and other cells survive. **(D)** Transplantation of breast cancer patient PBMC in the presence of human IL-4 (indicated by small orange circles). The immune suppressive characteristics are reflected in the mouse.

Laboratory mice engrafted with human cells or tissues enable researchers to study human biological responses in controlled environments. Hematopoietic stem cell (HSC)- and peripheral blood mononuclear cell (PBMC)-transplanted mice are two immune-humanized mouse systems based on cells or tissues transplanted into immunodeficient mice that have been reviewed in considerable detail previously (Kametani et al., 2019; Martinov et al., 2021; Chen et al., 2022) and are summarized in Figure 1. The human fetal lymphoid tissue-transplanted mouse system is not considered in this review because of ethical issues regarding the use of human fetal tissue and cells in chimeric research projects (Hyun, 2019). Here, we consider the main issues currently associated with HSC- and PBMC-transplanted mice and focus on a DDS and PBMC-engrafted humanized mouse model that we developed recently.

### 2.2 HSC-transplanted mice

HSCs are multipotent and can potentially develop into most blood immune cells if introduced into the appropriate molecular or physical environment (Bozhilov et al., 2023). Initial transplantation experiments of human HSCs into severely immunodeficient mice generated human lymphoid and myeloid cells, including human T and B cells localized in the peripheral circulation and xenogeneic environment of the mice (Figure 1A). However, erythroid lineage cells failed to engraft, and myeloid cell variations were limited in scope. Whereas most human B cells in humanized mice express CD5, a specific IgG antibody was not produced (Kametani et al., 2006; Kametani et al., 2016). These mice did not develop a germinal center with a structure composed of T, B or follicular dendritic cells, which are necessary for the development of antigen-specific antibodies. It was hypothesized that human T cell development in the mouse thymus required the human leukocyte antigen (HLA) system because the mouse major histocompatibility complex (MHC) disturbed the cognate interaction of human T cells and B cells. Consequently, MHC knockout (KO) mice were generated using an HLA class I/II transgenic system to express HLA molecules. Although HLA class I/II transgenic mice showed partially improved human humoral immune responses, these responses were weak and short-lasting (Suzuki et al., 2012; Jangalwe et al., 2016; Huang et al., 2019). One of the reasons for weak immune responses in these transgenic mice was the ineffective development of B cells, natural killer (NK) cells, innate lymphoid cells, and dendritic cells.

Various transgenic mouse strains expressing human cytokines and surface antigens along with more severely immunodeficient mouse strains were developed to support the differentiation of innate immune cells such as NK cells and dendritic cells (Magnotti and Marasco, 2018; Shan et al., 2022). These “next-generation humanized mice” were established based on the development of environmental factors such as IL-2, and stem cell- or myeloid-related cytokines such as stem cell factor or granulocyte-macrophage-colony stimulating factor (Martinov et al., 2021; Dalod and Scheu, 2022; Chen et al., 2023). For example, NSG-SGM3 mice, which expressed human stem cell factor, granulocyte-macrophage-colony stimulating factor, and IL-3 (Billerbeck et al., 2011), exhibited enhanced development of myeloid lineage cells with improved immune responses (Miller et al., 2022). However, these mice were highly susceptible to *Staphylococcus aureus* infection, and B cell fate did not improve dramatically (Hung et al., 2023). Immunodeficient mouse variants derived from human CD34^+^ HSCs have the potential to provide a human-like immune system with a higher number of naive T cells and improved engraftment in xenograft transplants (Bozhilov et al., 2023). However, technical issues continue to be difficult with human HSC engraftment and conditioning in the bone marrow niche of immunodeficient mice such as to establish an efficient human-like immune system may require more than 3 months to develop (Chen et al., 2023). Although most mouse strains supported the differentiation of hematopoietic cells transplanted from human HSCs compared to conventional immunodeficient mice, establishing a normal human immune environment in these mouse models requires further improvement.

### 2.3 PBMC-transplanted mice

PBMC-based humanized mice have some advantages over HSC-transplanted humanized mice because xenografted PBMCs are mature lymphocytes and myeloid cells that partially reflect donor immunity (Fukasaku et al., 2020; Yang et al., 2023). In contrast to HSC-transplanted mice, PBMC-transplanted mice do not contain granulocytes or erythroid cells and therefore are not engrafted in the mice. The main problem with the PBMC system is related to the activation of T cells against mouse tissues within a xenogeneic environment (Figure 1B) and the development of graft-versus-host disease (GVHD) resulting in short survival times for the experimental mice. T cell activation also decreases the survival and function of B cells, dendritic cells, and NK cells. Therefore, class I/class II MHC-KO mice were used to avoid recognition of the non-self by MHC molecules and GVHD (Ka et al., 2021). However, even if PBMCs were engrafted into these mice, they could not maintain B or myeloid cells (Yaguchi et al., 2018).

A CD47KO IL-2RgKO Rag2KO mouse strain (B6.129S Rag2tm1Fwa CD47tm1Fpl Il2rgtm1Wjl/J) that lacks Rag1, IL2rg or CD47 was developed for HIV infection analysis (Holguin et al., 2022). CD47, a widely expressed cellular receptor with immune regulatory functions, modulates cellular phagocytosis by macrophages, the transmigration of neutrophils, and the activation of dendritic, T, and B cells by interacting with its ligands. Thus, GVHD onset was delayed in CD47 KO mice, but almost all the surviving engrafted cells were T cells. Although these PBMC-transplanted mice avoided GVHD, they failed to maintain B cells that produce antibodies and that play an important role in almost all situations of immune homeostasis and diseases. We hypothesize that the inflammation caused by xenogeneic reaction induced the secretion of glucocorticoid, a stress hormone, which dampens the lymphocyte survival. While T cells might be rescued by the Lck-NF-kB signaling pathway through T cell activation in these mice (Harr et al., 2010), B cells might not recognize the survival signals, resulting in their death.

### 2.4 NOG-hIL-4-Tg humanized mouse strain for human T and B cell engraftment

We developed the NOG-hIL-4-Tg mouse strain, a new and simple human IL-4-transgenic mouse model, to produce tumor antigen-specific IgG antibodies by peptide vaccination (Kametani et al., 2017) (Figure 1C). Both human T and B cell engraftment into this mouse strain results in memory and effector phenotypes because the transgenic IL-4 function supports B cell survival. While IL-4 positive effects on B cell survival are reported, especially for memory formation (Chakma and Good-Jacobson, 2023), IL-4 together with IL-2 might impair glucocorticoid receptor phosphorylation, and decrease the immune suppression effect of glucocorticoid “*in vivo*” (Pazdrak et al., 2016). These transgenic mice did not upregulate glucocorticoid receptor expression in lymphocytes, increase the effect of glucocortocoids on immune suppression or impede B cells. We observed a negative correlation between the expression of glucocorticoid receptors and B cell number, and a positive correlation between B cell number and human IL-4 expression in this mouse system (Seki et al., 2018). In addition, healthy donor lymphocytes transplanted into hu-PBL hIL-4 NOG mice produced a HER2-antigen-specific humoral immune response, while those of patients with breast cancer were dampened (Ohno et al., 2021), (Figure 1D).

### 2.5 Humanized mice for evaluation of anticancer drug delivery systems

Drug delivery systems are designed to enhance the targeting, release, and effectiveness of cancer drugs with a focus on immune interactions. Table 1 lists some of the main immune-based DDSs used in contemporary research for immunotherapy, including monoclonal antibodies (checkpoint inhibitors), antibody-drug conjugates, nanoparticles, liposomes, and micelles.

**TABLE 1:** Main immune-based DDSs used in contemporary research.

Targeting cell	Method	Formula	Characteristics	References
DC and/or Tumor	DDS based on the chemical characteristics (for stability and diffusibility)	Liposome	Tumor accumulation and DC stimulation	Koshy et al. (2017), Ji et al. (2023)
		Endosome-destabilizing polymer vesicles		Wehbe et al. (2021)
		Lipid nanodiscs		Dane et al. (2022)
		Self-assembly of viral proteins	DC vaccine	Jneid et al. (2023)
		Exosome	DC vaccine	Yildirim et al. (2021), Wolfers, et al. (2001)
		Cell microparticles	PD-1 blocking	Wei et al. (2021), Takatsuka et al. (2023)
		Polymer nanodrug	DC polarization to Th1	Sun et al. (2022), Gu et al. (2019)
		Polymer nanoparticle	ICD induction by changing cold tumor to hot tumor	Wu et al. (2022), Li et al. (2020), Xiao et al. (2023)
Tumor	DDS based on immune-molecules	Antibody-drug conjugate	Tumor killing	Chau et al. (2019), Millul et al. (2021), Lowe et al. (2017), Li et al. (2016)
		Peptide-drug conjugate	Tumor killing	Zhou et al. (2021), Cooper et al. (2021), Zhang et al. (2023), Bennett et al. (2020)
	DDS based on cytotoxic cells	Bispecific T cell engagers, CAR-T, CAR-NK, TCR-T	Tumor killing	Ravi and Costa (2023), Sterner and Sterner (2021), Gong et al. (2021), Chen et al. (2023), Perales-Puchalt et al. (2019), Alijaj et al. (2020), Kim et al. (2020), Yang et al. (2023), Yu et al. (2017), Amissah et al. (2024)
	Oncolytic virus	Virus particle	Tumor killing	Shalhout et al. (2023), Man et al. (2022), Hamdan et al. (2021)

The use of monoclonal antibodies from different species to treat human infections and diseases can be harmful or fatal. For example, a human-mouse chimerically constructed superagonist anti-CD28 antibody, TGN1412, caused serious side effects in healthy volunteers when clinically tested, although it was considered safe in mice and apes (Attarwala, 2010). However, checkpoint inhibitors are a class of humanized monoclonal antibodies (mAbs) such as pembrolizumab (anti-PD-1), nivolumab (anti-PD-1), and atezolizumab (anti-PD-L1) that have been developed for use as immunotherapy drugs to block specific checkpoint proteins from binding with partner proteins, and therefore enhance the immune response against cancer cells (Marhelava et al., 2019). Other commonly used checkpoint mAbs target cytotoxic T-lymphocyte antigen 4 (CTLA-4), lymphocyte activation gene 3 (LAG-3), and T-cell immunoglobulin and mucin domain 3 (TIM-3) (Franzin et al., 2020). These cancer therapies can be evaluated for their effectiveness in triggering immune responses and their potential side effects safely in humanized mouse models. Antibody-drug conjugates, which are mAbs combined with cytotoxic drugs, also can be used in humanized mice to study the targeted killing of tumor cells while assessing their impact on human immune responses. Conventional immunodeficient mice such as NSG mice have been used successfully for the evaluation of CAR-T cells (Yang et al., 2023). However, they have not been used for the evaluation of antibodies and related DDS extensively because of the difficulty to control the xenogeneic reaction, which might disturb the evaluation. We used tumor-bearing PBL-NOG-hIL-4-Tg humanized mice and found that atezolizumab, an anti-PD-L1 mAb and a derivative cancer drug, are effective against the triple-negative breast cancer cell line MDA-MB-231 *in vivo* (Kametani, et al., 2023).

CAR-T cell therapy has shown remarkable success in treating certain types of leukemia and lymphoma, but challenges remain to be overcome, including managing side effects, such as cytokine release syndrome and neurotoxicity, as well as extending its effectiveness to solid tumors. Immunodeficient mice have been used to assess the effects of CAR-T cells on T cell lymphoma and other T cell malignancies (Chan et al., 2023; Michels et al., 2023). Although most experiments used non-humanized immunodeficient mice (Kobayashi et al., 2023), humanized NSG-SGM3 mice transplanted with human HSC were used to evaluate the efficacy of CAR-T cells in an allogeneic environment (Hu et al., 2023). While these mouse models engrafted and induced an anticancer effect, the molecular and cellular environments were not the same as those in patients. Immunodeficient mice that retain innate immune cell activity could decrease the effects of these human cell-based drugs. Thus, their efficiency might be overestimated because the immune system of patients is more complicated and contains regulatory T cells, regulatory B cells, and immunosuppressive myeloid cells, which differ among patients (Kumagai et al., 2024; Rassomakhina et al., 2024).

Although nanoparticles can carry anticancer drugs to specific sites, allowing targeted delivery and controlled release, their occasional immunotoxicity and health risks are concerning (Pondman et al., 2023). Liposomes and micelles are relatively safe lipid-based carriers engineered to encapsulate drugs and target specific cells or tissues. They protect drugs from rapid degradation and provide a form of controlled release that allow researchers to study their behavior. We used liposomes as a delivery system for a new cancer drug candidate, progesterone (P4), a pregnancy-related steroid hormone that exerts both immunoregulatory and anticancer effects (Kashiwagi et al., 2022). Because the P4 effect was induced at a concentration that was approximately 10 times higher than the physiological concentration in the placenta, we encapsulated P4 in liposomes at a lower concentration to target cancer and immune cells together. The Lipo-P4 DDS that we developed using the liposome encapsulation method enhanced the P4 effect and revealed that it was ten times more effective than free P4 in *in vitro* culture of various cancer cell lines (Seki et al., 2024). We also evaluated Lipo-P4 DDS using the humanized mouse system of PBMC transplanted into NOG-hIL-4-Tg mice (Kametani et al., 2023).

## 3 Reconstruction of a humanized immune environment for applications in cancer research

The immune environments of healthy donors and patients affected by cancer or autoimmune diseases may have different characteristics and responses that need to be assessed, such as cytokine imbalance, altered immune cell profiles, inflammatory responses, and immune checkpoint dysregulation. Therefore, it is desirable for humanized mice to reconstruct the characteristic responses of patient immune environments when they are transplanted with PBMCs from patients affected by cancer or autoimmune diseases. These humanized mouse models help evaluate the effectiveness, safety, and immune responses of new drugs and DDSs; as well as interactions between the human immune system and cancer. The patient-derived xenograft (PDX) model, which is the transplantation of human cancer tissues into humanized mice, is a popular system to reconstruct the environment of cancer patients (Jin et al., 2023; Liu et al., 2023). While PDX models have many advantages, they also have limitations. For example, transplantation of the PBMC together with PDX to reconstruct a cancer immune condition often induce GVHD-based cancer rejection, which might result in an over-estimation of the anticancer drug effect. Moreover, B cell effects cannot be evaluated in these systems.

Tumor-bearing PBL-NOG-hIL-4-Tg mice, which suppress GVHD, demonstrated the effectiveness of atezolizumab, an anti-PD-L1 monoclonal antibody, and a progesterone cancer drug against the triple-negative breast cancer cell line MDA-MB-231 *in vivo* (Kametani et al., 2023). The T cells engrafted in the mouse secreted both Th1/Th2 cytokines, suggesting that immunity did not shift to Th2, and lymphocytes expressed their normal function. Therefore, this system overcomes several important problems. However, because the number of transplanted PBMCs is limited, the cellular repertoire of each mouse transplanted with the same donor PBMC may vary, resulting in different responses to the same antigen. Because most engrafted lymphocytes showed a memory phenotype, and very few naïve T and B cells were observed in the mouse lymphoid organs, these lymphocytes are probably maintained by homeostatic proliferation (Jameson, 2005; Baliu-Piqué et al., 2022). These mice did not develop follicular dendritic cells, which are necessary for affinity maturation. Although the mice produced antigen-specific antibodies, affinity maturation was not as extensive as that in wild-type mice or humans. In future studies, peripheral PBMCs from sensitized donors should be transplanted into mice to produce high-affinity antibodies.

The anticancer effect of the liposome P4 encapsulation DDS (Lipo-P4) was established successfully in our PBL-NOG-hIL-4-Tg mice *in vivo* (Kametani et al., 2023). Interestingly, the DDS effect was not limited to cancer cells, but also involved immune cells such as T cells and B cells. The proportion of CD8^+^ cytotoxic T cells increased, whereas that of B cells decreased in Lipo-P4-treated mice. Most of the engrafted T cells were central memory T cells, and Treg cells were not increased. We also used an antibody against PD-L1 that is frequently expressed in cancer cells, activated T cells, and professional antigen-presenting cells (Chen et al., 2016). Our studies suggest that the Lipo-P4 affects both cancer and immune cells and improves the anti-PD-L1 antibody effect against PD-L1 that is expressed on cancer cells and highly activated lymphocytes. In addition, the immune-related adverse events (IRAEs) are not severe because the accumulation of human lymphocytes in the mouse lung and liver were at a very low level. However, further ongoing evaluation including the possibility of xenogeneic inflammation is still needed. In addition, IRAEs may be related to progesterone receptor expression on the plasma membrane or mitochondrial membrane, and not only in the nucleus. If anticancer drugs have multiple receptors both inside and outside the nucleus or organelles, the delivery system can be fine-tuned to target specific receptors.

In a different strategy for reconstructing cancer immunity of humanized mice, we transplanted the PBMCs of breast cancer patients. In this system, Nivolmab treatment against PD-1 suppressed the B cell response and increase T cell numbers, which might reflect the cancer immune response of the patients (Ohno et al., 2021). We need further detailed comparisons to better differentiate between the patient and the humanized mouse response to the patient’s breast cancer cells.

## 4 Discussion

Immunotherapy-related DDSs in humanized mice models represent a dynamic and evolving area of cancer research. In this minireview, we focused on a PBMC-engrafted humanized mouse model, the NOG-hIL-4-Tg mouse strain, that we developed for evaluating immune-related, anticancer drug delivery systems. Our model has some important advantages over other systems such as the HSC mouse model and those listed and referenced in Table 1. While other humanized mouse system developments have focused mostly on the T cell response, we have shown that the NOG-hIL-4-Tg mouse model has benefits in reconstructing the humoral immunity of donors. PBMC transplantation can reconstruct the environment of mature T cells and B cells, and the proportion of the subsets are the same as the donor’s. If the cancer patient’s PBMCs are transplanted, the mouse can quickly reconstruct the individual patients’ immune environment. In contrast, the HSC-transplanted mouse needs more than 3 months to develop mature human T cells and B cells, while the PBMC-transplanted mouse can be used immediately after transplantation. Also, the reconstructed immune cell subset in the HSC-transplanted mouse is not the same as HSC donors, and the HSC-transplanted humanized mouse cannot mimic the patient’s immunity. The induction of GVHD, by which the normal immune reaction is disrupted, is a limitation in some PBMC-transplanted mice models. However, NOG-hIL-4-Tg mice have suppressed GVHD and can maintain B cells because of the IL-4 effect. Moreover, IL-4 suppresses glucocorticoid receptor signal on B cells, which is negatively correlated with the B cell engraftment.

Finally, our Lipo-P4 DSS has two functions, one is an anticancer effect, and the other is immune regulation. Progesterone (P4) increases the number of cytotoxic T cells and downregulates the effector function, which is fine-tuned to avoid immune-related adverse events. Moreover, progesterone does not dampen the patient’s immunity as severely as glucocorticoid does. By integrating the DSS with human-like immune responses and cancer characteristics within our humanized mouse model, we and other researchers can develop and test new therapies that are more effective and targeted in order to safely advance personalized and immuno-oncology treatments.
